# Exploration of Roles and Contribution of Spiritual Care Practitioners in Mental Health: An Australian Study

**DOI:** 10.1007/s10943-024-02214-1

**Published:** 2025-01-25

**Authors:** Shikha Malviya, Jenny Greenham

**Affiliations:** 1 Occupational Therapy, School of Health and Medical Sciences, University of Southern Queensland, Toowoomba, QLD 4350 Australia; 2 Spiritual Health Association, Melbourne, VIC Australia

**Keywords:** Spiritual care, Spiritual care practitioners, Mental health, Spiritual needs, Chaplains

## Abstract

Mental health is inherently multidimensional, requiring a holistic approach to intervention that integrates various aspects of an individual’s well-being. Spirituality, a vital component of mental health, remains under addressed in Australian mental healthcare. Spiritual care practitioners may play a key role in addressing spiritual needs in mental healthcare; however, their roles and contributions in this context remain unexplored in the extant literature. Bridging this gap, this study explores the potential role of spiritual care practitioners within mental health context. Using a qualitative research approach, this study engaged eight experienced spiritual care practitioners working in various mental health settings across Australia (n = 8). Through reflexive thematic analysis, the study identified and examined the practitioners’ perspectives on their roles and contributions. The findings were summarised in three overarching themes: (1)  Core values; (2) Unique contributions in mental health; and (3) Spiritual care practitioners in the mental health system. The study’s findings suggest that by employing a person-centred approach, spiritual care practitioners can play a crucial role in mental health assessments and interventions. Their contributions include providing insights rooted in clients’ unique spiritual beliefs, aiding in the discernment between spiritual experiences and psychopathological symptoms, advocating for clients’ spiritual needs, and supporting the education of mental health professionals. The study also highlights the need for professional recognition of spiritual care practitioners and their greater integration within the mental health system.

## Introduction

In 2019, approximately 970 million people worldwide were living with a mental health disorder (World Health Organisation [WHO], 2022). In Australia, more than two in five individuals aged 16–85 years (43.7% or 8.6 million people) have experienced a mental health concern at some point in their lives (National Study of Mental Health and Wellbeing, 2023). Given the complexity and prevalence of mental health disorders, a holistic approach to intervention is essential to address the multidimensional nature of mental health (WHO, 2022). Spirituality, recognised as an integral aspect of mental health (WHO, 2022), is associated with numerous benefits, including positive mental health outcomes, enhanced resilience, and overall well-being (Bonelli & Koening 2013; Peres et al., 2007; Fry, 2000; Geneva Charter for Wellbeing, 2021).

While there are several definitions of spirituality in the literature, two key perspectives provide valuable insight. Puchalski et al., (2014) define spirituality as “the aspect of humanity that refers to the way individuals seek and express meaning and purpose and the way they experience their connectedness to the moment, to self, to others, to nature, and to the significant or sacred” (p. 643). This highlights the deeply personal and interconnected experience that spirituality offers, encompassing not only individual reflection, but also connection to others and the world around us. Similarly, Koenig (2012) views spirituality as “the personal quest for understanding answers to ultimate questions about life, about meaning, and about relationship to the sacred or transcendent, which may (or may not) lead to or arise from the development of religious rituals and the formation of community” (p. 2). While the later definition broadens the scope, acknowledging spirituality as a personal pursuit of existential meaning, independent of formal religion, both emphasises the importance of addressing spirituality within mental healthcare.

In Australia, despite a decline in overall religious affiliation (Australian Bureau of Statistics [ABS], 2022), a national survey revealed that nearly 70% of Australians hold religious or spiritual beliefs (McCrindle, 2017). Additionally, one in two Australians perceive spirituality as closely linked to their mental well-being (McCrindle, 2017). Studies further suggest that many Australians expect healthcare providers to explore and integrate spirituality as part of their overall care (Best et al., 2014, 2015; Hilbers et al., 2010). Incorporating spirituality into mental healthcare, particularly for Australians from culturally and linguistically diverse (CALD) backgrounds, is important, as this population often draws mental health support from their religious beliefs (Malviya, 2023). Moreover, spiritual practices are increasingly recognised for their mental health benefits (Malviya et al., 2022a, 2022b; Malviya, 2023, 2024; Marek & Lisiecki, 2024), with some of these practices being considered potential therapeutic interventions by mental health professionals in Australia (Malviya et al., 2022c). However, despite substantial evidence linking spirituality to positive mental health outcomes and a clear preference among Australians for its integration into care, spiritual aspects are frequently overlooked within the mental healthcare system in Australia (He & Petrakis, 2023; Malviya, 2023).

One key reason for this oversight is the lack of clear role demarcation regarding who within the multidisciplinary mental health team is responsible for assessing and supporting spiritual needs (So et al., 2023). While several official documents, such as the National Safety and Quality Health Service Standards (2018) and the National Framework for Recovery-Oriented Mental Health Services (2022), emphasise the inclusion of spirituality (sometimes conflated with culture) in healthcare, it remains unclear which health professionals are equipped to address spirituality within mental health contexts (Jones et al., 2021a). This ambiguity is further compounded by a general lack of knowledge and confidence among mental health clinicians in assessing and intervening on spiritual aspects of care (Best et al., 2016; Jones et al., 2021a; Ledger & Bowler, 2013; Malviya et al., 2022c; So et al., 2023).

One potential contributing factor to this gap is that spirituality is not typically a core component of training for most medical and allied health professionals (Jones et al., 2021a; Roger et al., 2019). Consequently, many health professionals lack the skills necessary to address spiritual aspects of care and often feel uncomfortable and uncertain in doing so (Drew et al., 2021). Additionally, although healthcare professionals are expected to include spiritual care in their roles (Jones et al., 2021a, 2021b; Ledger & Bowler, 2013), barriers such as competing healthcare priorities, negative perceptions of spiritual care, and inadequate staff training hinder the implementation of such practices (Ledger & Bowler, 2013). This highlights the need for specialised spiritual care practitioners with the knowledge and experience to deliver focused spiritual care (Balboni et al., 2014; Puchalski et al. 2014).

Historically, clergy played a primary role in providing spiritual care within organisations and communities before hospitals formally employed spiritual care practitioners (Carey et al., 2016, Verkamp 2006). In contemporary healthcare settings, clergy and chaplains have emerged as trained professionals in addressing the spiritual needs of patients (Handzo et al., 2014; Carey and Rumbold 2015). While clergy are religious leaders ordained within specific faith traditions, chaplains—or spiritual care practitioners—are trained to provide spiritual support (sometimes also referred as pastoral care) in diverse contexts, often serving individuals from various faiths or none (Aiken, 2022; Spiritual Care Australia [SCA], 2013). The terms “pastoral care” and “spiritual care” are sometimes used interchangeably in the literature, though there are subtle differences (Carey et al., 2024).

Pastoral care has traditionally been rooted in religious contexts, particularly Christianity, though its scope has broadened over time. Carey and Cohen (2015) describe pastoral care as “a person-centred, holistic approach to care that complements the care offered by other helping disciplines while paying particular attention to spiritual care” (p. 1). Spiritual care, however, is often viewed as a more inclusive concept that transcends religious boundaries. Puchalski et al., (2014) define spiritual care as care that “recognises and responds to the needs of the human spirit when faced with trauma, ill health, or sadness, and can include the need for meaning, for self-worth, to express oneself, for faith support, perhaps for rites or prayer or sacrament, or simply for a sensitive listener” (p. 643). While both forms of care share similarities, pastoral care is typically seen as encompassing a broader range of aspects, potentially including faith-specific spiritual, emotional, and relational elements, whereas spiritual care focuses more specifically on addressing the spiritual dimensions of well-being.

Working in various settings such as schools, defence, hospitals, and prisons, chaplains “fulfil the role of retaining that which is considered sacred (For e.g. values, beliefs, ethical principles and morality), and provide support, counselling and education plus various rituals and rites of passage in order to ensure holistic care” (Carey & Rumbold, 2015, p. 1222). In a systematic review of observational and experimental studies regarding the use of chaplains in the hospital setting, spiritual care was correlated with increased patients’ satisfaction (Kirchoff et al., 2021). The care given by chaplains was also associated with spiritual well-being and better quality of life (Kirchoff et al., 2021). Since spirituality is a crucial aspect of mental health, chaplains play a significant role in healthcare by addressing spiritual well-being (Balboni et al., 2022; Carey et al., 2015; Holmes [Bibr CR41][Bibr CR78],b).  In recent literature, the terms “chaplain” and “spiritual care practitioner” are often used interchangeably (Best et al., 2022; Standards of Practice, Spiritual Care Australia [SCA], 2013). However, for the purposes of this study, we will use the term spiritual care practitioners.

Although in the past, faith-based interventions in healthcare have been valuable, given the rapidly changing religious landscape of Australia (Australian Bureau of Australia (ABS), 2022), there is an emerging need for spiritual care practitioners who can provide spiritual support irrespective of the religious or non-religious background of the recipient of care. Working in different settings such as hospitals, community settings, schools, prisons and defence, spiritual care practitioners provide valuable mental health support to clients, care givers, and other staff (Best et al., 2023). Spiritual care practitioners aim to provide spiritual care in response to clients’ unique needs and religious/spiritual background (SCA, 2013). Though some spiritual care practitioners may be trained as religious chaplains for a particular faith (and may also provide faith-specific spiritual care), this is not a requirement to become a spiritual care practitioner (Spiritual Care Australia [SCA], 2013). Thus, spiritual care practitioners are trained professionals capable of delivering client-cantered spiritual support in both religious and non-religious contexts (Layson et al., 2023).

A qualitative study with chaplains in New Zealand explored their roles in the mental health context (Carey & Medico, 2013). The research revealed that these chaplains play various roles, including conducting pastoral assessments, providing emotional and spiritual support, offering counselling services, delivering educational interventions, and performing rituals for both clients and their families (Carey & Medico, 2013). However, participating chaplains also noted challenges such as limited client access, poor communication, and a lack of recognition of their contributions (Carey & Medico, 2013). Noting several implications for healthcare institutions and government responsibilities, the authors of the study identified the need for further research (Carey & Medico, 2013).

In Australia, limited studies have explored the awareness among mental health professionals of the role of spiritual care practitioners (e.g. Jones et al., 2022a). A comprehensive review by Carey et al. (2016) outlined the ways that chaplains can address mental health concerns linked to moral injury among defence personnel. While the review provides important insights into the role of chaplains in addressing moral injury, it did  not  seek to address their role within the broader context of mental health context. As such, despite the invaluable contributions of spiritual care practitioners, their roles and responsibilities, particularly within the mental health field in the Australian context, remain unclear (Best et al., 2021).

This study aims to explore the roles and contributions of spiritual care practitioners in mental health without focusing on specific conditions or populations. By examining the perspectives of experienced practitioners, this study seeks to provide preliminary insights into their overarching contributions, potentially enhancing the current evidence base and raising awareness of their critical roles in mental healthcare.

## Methodology

### Research Design

A qualitative research approach was selected to generate insights and explore the perspectives of research participants (Castellan, 2010). Given the nature of the research enquiry and the nuanced information required to achieve the study’s aim, this approach was deemed most appropriate. Focus groups are recognised as a cost-effective method of data collection, particularly for exploring less well-known phenomena (Bertrand et al., 1992). Due to the paucity of research on the role and contributions of spiritual care practitioners in mental health, focus groups were chosen as the primary method for data collection. This study, therefore, includes a focus group of experienced spiritual care practitioners working in various mental health settings.

### Compliance with Ethical Standards

The ethics approval was obtained from Darling Downs Health Human Research Ethics Committee (approval number- HREC/2023/QTDD/98119) prior to the commencement of the study. Participants consented to participate in the study, and publication of the research based on the collected data, using pseudonyms.

### Participants

#### Sample Size and Eligibility

Purposeful sampling increases the likelihood of obtaining rich, nuanced data (Palinkas et al., 2015). For this study, a convenient purposeful sample of spiritual care practitioners with a minimum of two years’ experience working in mental health was recruited. In qualitative research, emphasis is placed on the relevance and quality of the knowledge contributed, meaning that even studies with small sample sizes can provide valuable insights (Malterud et al., 2016, Boddy, 2016).

Participants were recruited by SM and JG through the Mental Health Network Group of the Spiritual Health Association (SHA, Melbourne, Victoria). Although no formal screening assessments were conducted, participants were required to declare in the consent form that they did not have any condition that could impair their ability to share their views.

#### Recruitment

After ethics approval, researchers SM and JG emailed potential participants with initial project details, requesting responses within one week. Those interested received an information sheet/consent form and were asked to return the signed form within two weeks. Recruitment began upon receiving written consent, after which researchers scheduled a convenient day/time for the focus group session. The focus group was conducted over Zoom at a mutually agreed time to address the research questions.

### Data Collection

The demographic data were collected through individual emails prior to the focus group session. The duration of the focus group discussion session was 90 min. A set of prompt questions were used to facilitate the session. The prompt questions are listed in Table 1. Experienced participants were able to provide their perspective regarding the roles and contribution of spiritual care practitioners in mental health. The discussion was audio recorded and transcribed for data analysis using NVivo (Dhakal, 2022). The primary researcher SM checked the transcribed data for any potential transcription error. At this stage, data were de-identified, and participants were allocated random letters, which were used for data analysis to maintain their anonymity.

**Table 1:** Prompt questions to be used in focus group discussion

How have you contributed to the assessment of patients/clients in mental health care?
What interventions do you offer or have offered to support patients/clients in mental health care? (What’s the connection between your assessment & intervention?)
Please share your perspective about the changes/outcomes you see due to your intervention? Is there any specific feedback you have received from colleagues and or patients/clients?
What is the most important change you could recommend to advance mental health spiritual care?

### Data Analysis

Qualitative data are comprehensive, subjective, and often interlaced with multiple concepts and ideas (King, 2004). Before analysis of data, participants had the chance to add further comment/feedback to the content if they wished to do so. Transcribed data were then coded and analysed using thematic analysis. Thematic analysis is a useful method for examining the data, highlighting similarities and differences, and generating unanticipated insights through identification of common themes (King, 2004). Reflexive thematic analysis (TA) is a method for analysing, developing, and interpreting patterns across qualitative data (Braun & Clarke, 2019). Reflexive TA was the chosen method of data analysis due to its flexibility and accessibility (Braun & Clarke, 2019).

The data analysis was conducted in six steps (Braun & Clarke, 2019), collaboratively by both authors. In step one, authors read and familiarised themselves with collected data, followed by line-by-line coding of the transcripts in step two. In step three, codes were organised into tentative subthemes and themes. After reviewing these subthemes and themes in step four, they were labelled and merged into overarching themes in step five. Step six involved extracting quotes that best described subthemes.

## Findings

### Participants

A total of eight spiritual care practitioners participated in the study, all of whom had experience working in mental health settings as spiritual care practitioners. While details of their current healthcare settings were not collected, all participants had experience across diverse mental health environments. The participants’ ages ranged from 35 to 64 years, and their years of experience as spiritual care practitioners ranged from 2 to 25 years. Demographic information is presented in Table 2.

**Table 2:** Demographics of participants

Serial Number	Participant	Age/Sex	Religious/spiritual affiliation	Number of years of experience as spiritual care practitioner
1	S	51/Female	No religion	6 years
2	H	57/Female	Anglican	25 years
3	M	59/Male	No religion	10 years
4	L	64/Female	Catholic	10 years
5	T	35/Female	Christian	6 years
6	R	54/Female	Catholic	3 years
7	J	57/Female	Catholic	9 years
8	A	63/Male	Charismatic (Christian)	2 years

### Themes and Subthemes

**Fig. 1:**
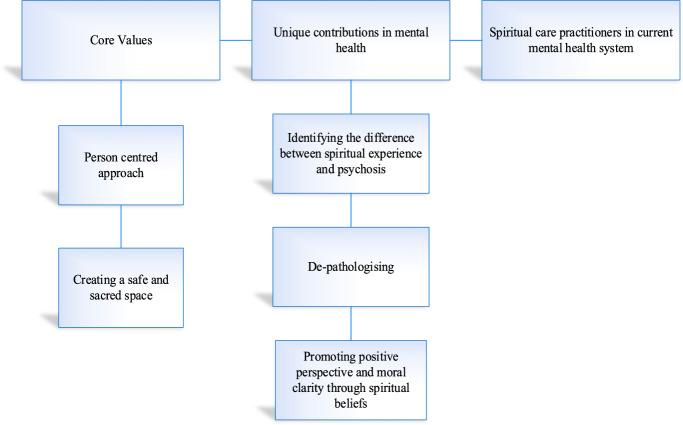
Themes and subthemes

The data were initially coded into 45 codes. These codes were iteratively grouped into subthemes, which were then conceptualised and organised under three overarching themes: (1) Core values, (2) Unique contributions to mental health, and (3) Spiritual care practitioners in the current mental health system. The first theme comprises two subthemes, and the second theme includes three subthemes. The finalised themes and subthemes are illustrated in Fig. 1 and further described below, accompanied by illustrative quotes from participants.

#### Core Values

Participants highlighted key professional core values that guided their interventions in mental healthcare. These are summarised in the following two subthemes.

- Person Centred Approach

Participants emphasised that their interventions were always guided by the needs and preferences of their clients, with clients often determining the level and form of spiritual care they required. For instance, M shared,

*I strive to offer person-centred care, genuine person-centred care, honouring the way and the timing of the person’s telling their lives narrative rather than driving an agenda of my own of fixing that person. My aim is to support the person when they’re exploring their relationship with their life challenges and past experience of trauma and hurt.*

Similarly, T added,

*I come from a person-centred perspective, so the conversations and the sessions are led by the client in terms of exploring their goals their desires and their agenda, so allowing that person to guide the conversation and meaning to emerge.*

In addition to providing person-centred care, participants stressed the importance of non-judgmental, empathetic presence and a genuine interest in the person’s well-being through active listening. For example, T elaborated,

*I do value letting the person lead the session and following their agenda and us truly listening to what their story is, how they are narrating their story, and listening for certain feelings and spiritual themes.*

M further described this client-centred approach in the context of assessment, stating, “*It’s really about sitting beside the person and observe along the way rather than coming from the attitude of scrutiny and measurement”.*

Although non-judgmental and person-centred approaches are common across many mental health professions, participants highlighted that these approaches are at the core of their work as spiritual care practitioners. For example, R said,

*When we talk about being non-judgmental, I think every professional in the ward would argue the same thing. But I think* [with] *spiritual care comes at it differently um I think we take it seriously because it’s our core* [values]*.*

b) Creating a Safe and Sacred Space

In the potentially stressful atmosphere of the mental health setting/unit, participants placed great value on creating a safe and sacred space for their client throughout their interactions and interventions. While discussing about creating safe space, H mentioned, “*When everybody’s holding the floor as close as they can, people feel human when they talk to me, they don’t feel like a patient, they feel safe and respected”.* Another participant L extended this by describing how she literally created a sacred space in a mental health unit,

*I’ve created a sacred space where I have different multi faith resources. They can come and spend quiet time there. I’ve have let them all know that that everyone’s accepted. I have a group called “space for grace”, where women get together, and I do a reflection for them*.

#### Unique Contributions in Mental Health

Participants shared several ways they contributed to mental healthcare. Some of the contributions that were unique to their profession are consolidated in the following three subthemes.

- Identifying the difference between spiritual experience and psychosis

One of the unique contributions, participants mentioned was about the insight they offered in discerning the difference between spiritual experience and psychotic phenomenon. H mentioned,

*There have been times when I helped the team see the difference between a religious experience and a psychotic experience. We can help them* [mental health professionals] *see the difference. In the spiritual/religious experience, it is like giving, and it opens up whereas, when it’s psychotic thinking, it basically blocks everything and keeps people going through the washing machine cycle.*

Another participant S highlighted her contribution, when religious beliefs were mistaken as a feature of psychotic episode. S shared,

*I see my role in helping to identify the difference between psychosis and spiritual experience within the faith context, where the faith had elements that were seen as psychosis. But within the faith context, and the culture of that particular faith, were quite normal beliefs. For example, a person I helped, within his faith which was Pentecostal, that* [client’s thought content]* was consistent with his faith, it made sense within that faith view, though that seemed to be concerning for everyone.*

While acknowledging that discerning the difference between these two phenomena is extremely difficult, participant T highlighted her role to alert the treating team, lest an element of psychosis surfaced while exploring spiritual themes,

*It takes very skilled practitioner to see the difference between genuine spiritual emergency and psychotic episode. It can be mishandled if someone is experiencing a psychotic episode, but it’s treated like a spiritual emergency or vice versa. If someone’s experiencing a spiritual emergency and it’s treated like a psychotic episode, then it adds to the trauma. If spiritual carer believes there’s risk of a psychotic episode, they discuss with the multidisciplinary team to ensure that the client’s health is addressed.*

b) De-Pathologising

Participants discussed how, in mental health care, what appears to be irrational or fragmented may have deep spiritual significance for that person. Several participants shared their experience of how they contributed to identifying spiritual elements in what were deemed (by mental health professionals) as part of mental health symptoms. Through an elaborate example, participant S explained how she could help her client in identifying the inherent spiritual significance,

*There was a symbolic nature to what she* [S’s client]* was describing that was deeply meaningful to her and that by getting to that and not just pathologising that helped her organise her experience and come to deeper understanding of what was happening for her, kind of relinking of herself rather than a further fragmentation.*

Participants highlighted that, in mental health, sometimes complex accounts that may be considered as a part of psychopathology, require deeper exploration. They viewed their role as crucial in exploring such accounts, which could contribute to more accurate diagnosis and care. H illustrated this with an example,

*I think I’ve been good at de-pathologising.* [explains the example]*, We’re talking about a person, not a checklist or a diagnosis. So very much respecting the whole person rather than trying to nail it into a diagnosis and if a person has condition X, then we do Y without actually looking at what does this person need.*

c) Promoting Positive Perspective and Moral Clarity Through Spiritual Beliefs

Participants shared that they helped their clients in changing their unhelpful (negative) outlook to a helpful (positive) one by employing the clients’ unique spiritual beliefs, which thereby helped in their mental health. For example, participant J explained,

*I will often take people to their own religion, to their own scriptures so depending on where they come from, I help them define things* [from their spiritual perspective]*, which will take them away from it* [stress]*, into a delight.*

Additionally, by using above strategy, some participants seemed to support clients addressing the mental conflict related to the notion of morality by offering context and alternative perspectives grounded in clients’ own spiritual beliefs. For example, J shared, “*I find a lot of people with mental illness, what’s happening in their head they’re bad or they’re wrong and so our role is to help them understand the morality is about action not about the voices”.* On the same note, another participant L shared an example,

*One client was telling me about the terrible thing that happened to her, and she was carrying this Catholic guilt for a long period of time, thinking that she was not worthy. With my knowledge* [about Christianity], *I was able to bring up parables like the parable of the prodigal son and let her see for herself that God was not this judgmental God, but an unconditional loving God and she saw that, and then she slowly came around. So, from that perspective I think she was able to let go a little bit of that guilt.*

#### Spiritual Care Practitioners in the Mental Health System

Participants discussed their role in advocating for clients’ spiritual needs, particularly in a mental health system, moving from a medical model to holistic person-centred care. M reflected,

*As mental healthcare shifts from the addressing symptoms as indicators of pathology to listening to a person’s narrative as an opportunity for learning, growth, healing, so focussing on truly person-centred care, I see, there is strong central relationship between mental health and the spiritual care workforce.*

With strong focus on holistic care of mental health, participants identified the importance of their role as an advocate for their clients, especially because within a mental health context, clients’ request of such spiritual interventions may be dismissed on account of their mental state. While discussing an example of a client of Pentecostal background who wanted to meet their pastor for a spiritual intervention, S mentioned,

*So, the Pentecostal fellow* [S’s client]*, he really wanted to connect to a pastor. For him he did not think he had mental health issues, he felt they were deeply spiritual issues. I talked a lot with the psychiatrist about allowing him to have that intervention. But they* [mental health team]* thought it would feed his unhealthy obsession.*

Despite the essential contributions of spiritual care practitioners to mental health, several participants expressed concerns about insufficient funding and the lack of respect for their role within the multidisciplinary team. For example, one participant R shared, “*My department is not funded for spiritual care in mental health, so I feel constantly under threat”.* Another participant A added, “*In America they* [spiritual care practitioners] *are seen as a profession and we are not seen as a profession here* [in Australia].

Several participants also noted the lack of respect of their role among the multidisciplinary team. Participant S observed that it could be due to the lack of understanding of their role,

*I think it’s easier to get adversarial because we hold such a different understanding of what’s happening, but the medical profession is holding a very important piece and it is what it is. So, I feel like building those aspects of mutual respect by respecting and also appreciating what you’re holding there and what we’re holding here, and this is what I’m bringing to the table. So that there can be this growing shared understanding about the different pieces that are being held and yes, the differences but also where they intersect.*

As such, participants emphasised the need for continuous education of staff and multidisciplinary teams. This is especially true as by default spiritual care can be understood as religious care, hence not relevant for non-religious clients. T mentioned,

*It is important to educate staff members and colleagues at the workplace around what spiritual care is and how it can support mental health because that tends to be a huge barrier for staff and for patients. A lack of understanding as to what it is and especially with the history of pastoral care and how it can have religious connotations, it can therefore be a lot more specific for people and if people aren’t religious, they think that it won’t be beneficial to them, so education is a huge.*

Additional to educating staff, some participants also recognised their role in supporting mental health staff by providing them spiritual support, for example L shared,

*When we lost a patient on the ward, the staff came up and said can we have a little ritual, not necessarily a particular faith ritual but the community kind of a ritual to honor her* [deceased client]*. So, even the staff looking to us for direction, so we had a beautiful ritual to honour her life.*

## Discussion

This study set out to explore the perspective of experienced spiritual care practitioners regarding their roles and contributions in mental health. Participants highlighted their views regarding professional values, their unique contributions to mental healthcare, and the necessity for better professional recognition. Participants also recognised the need for professional identification and emphasised the importance of education of mental health professionals about the unique role spiritual care practitioners play in mental health.

Spiritual care has long been acknowledged as integral to mental health (Koenig, 2009; WHO, 2022), but challenges persist in determining which professional group should take the lead. In most western countries, chaplains and spiritual care providers are formally integrated into healthcare systems (Brady et al., 2021; Best et al., 2020). In Australia, despite the recognition of its value, spiritual care is inconsistently provided within mental health settings. This is because mental health professionals generally do not receive adequate training (Jones et al., 2021a), and currently, the professional with responsibility to provide that care has not been formally identified. Due to this, spiritual care is often overlooked, and neglected in mental healthcare (Milner et al., 2019).

Participants in this study pointed out that spiritual care practitioners possess specialised skills and knowledge that could be harnessed to address spiritual needs in mental health contexts. Findings suggest that spiritual care practitioners may offer valuable insights into various aspects of mental health assessments and can be instrumental in providing spiritual care interventions. Though other professionals such as nurses (McSherry & Jamieson, 2011, 2013), occupational therapists (Morris, 2013), and social workers (Francoeur et al., 2016) place importance of spiritual care to a certain extent, it is not always translated in their clinical practice (Jones et al., 2021a; Ledger & Bowler, 2013). In fact, literature suggests that mental health professionals often feel nervous in addressing the sensitive subject of one’s spirituality (Ledger & Bowler, 2013; McSherry & Jamieson, 2013), and feel reluctant in providing spiritual care to their clients (Selby et al., 2016). Spiritual care practitioners, with their empathic, person-centred approach, provide a safe and non-judgmental space where clients can explore and meet their spiritual needs. As such, including spiritual care practitioners in multidisciplinary teams can address this gap and ensure that the spiritual aspect of mental health care is not neglected.

Spiritual beliefs and practices often influence the clinical presentation of mental health disorders, particularly in cases involving psychosis (Koenig et al., 2020). Challenges in differentiating between spiritual experiences and psychopathology are well-documented in the literature (for example, Johnson & Friedman 2008; Sperry & Shafranske, 2005). For people from CALD backgrounds, these challenges are exacerbated by mental health professionals’ lack of understanding of their religious and spiritual beliefs, often leading to misdiagnosis (Liang et al., 2016). It is argued that with thorough knowledge of various forms of spiritual beliefs and experiences, openness, and sensitivity, such misdiagnosis may be prevented (Johnson & Friedman 2008). Some experts in the field further suggested that consultation with spiritual experts in contextualising these experiences may mitigate chances of misdiagnosis, and contribute to planning suitable interventions (Johnson & Friedman 2008; Koenig et al., 2020). Findings of our study suggest that in countries with diverse populations, like Australia, the inclusion of spiritual care practitioners who are trained in navigating various spiritual beliefs and practices can help mitigate these risks. These practitioners can support mental health professionals by offering insight into the spiritual dimensions of a client’s experience, potentially preventing misdiagnosis and informing more tailored interventions (Johnson & Friedman 2008; Koenig et al., 2020).

The evolving nature of spirituality in contemporary society demands a more inclusive model of spiritual care. As the number of people identifying as spiritual but not religious (SBNR) increases, particularly in Western contexts (ABS, 2022), spiritual care needs to be adapted. Some scholars in the field argue that moving away from a traditional faith-based model may render the role of spiritual care professionals superfluous, as other health professionals provide “secular” care (Layson et al., 2023, p. 1492). However, our findings suggest that spiritual care practitioners may help meet the spiritual needs of individuals from diverse belief systems by offering flexible, person-centred care that extends beyond conventional religious frameworks. For instance, in an Australian qualitative study with 16 spiritual care practitioners, participants described “broad conceptualisations of spirituality, linking it with life, meaning, and connectedness” (Best et al., 2023, p. 1482). Such broader frameworks are becoming more relevant as Australia becomes increasingly diverse, with more individuals identifying as SBNR (ABS, 2022). This trend points to a need for a model of spiritual care that can accommodate a variety of worldviews, including secular spiritualities (Layson et al., 2023).

Despite the contributions of spiritual care practitioners, they remain under-recognised in healthcare, particularly in mental health. This aligns with existing literature pointing to misunderstandings of their role (Purvis et al., 2018; van de Geer et al., 2017). Participants in our study suggested that educating other healthcare professionals about the role of spiritual care practitioners might foster greater awareness and collaboration within multidisciplinary teams (Best et al., 2020). Previous research indicates that spiritual care practitioners often play a role in education, modelling spiritual care for other professionals and demonstrating ways to incorporate it into clinical practice (Jones et al., 2021a; 2022a; 2022b). In their systematic review, Jones et al. (2021a) concluded that spiritual care training for healthcare professionals may include interactive approaches such as case studies, group discussions, and role-play exercises. The review’s findings further emphasised the value of self-reflection and exploration of one’s own spirituality (Jones et al., 2021a). Using these approaches, spiritual care practitioners may provide education and training to mental health professionals about the inclusion of spiritual care in their practice.

However, there are significant barriers to integrating spiritual care practitioners fully within mental healthcare, primarily due to limited funding and professional recognition. In a recent cross-sectional survey of 897 Australian inpatients, nearly 32% identified spiritual care practitioners (termed pastoral care staff in the study) as a preferred resource to address their spiritual needs (Best et al., 2024). Yet, despite these patient preferences, there remains a lack of investment for positions dedicated to spiritual care within mental health services.

In Australia, spiritual care practitioners are often employed on a contractual basis by religious organisations rather than directly by healthcare organisations (Best et al., 2022), limiting their capacity for consistent contributions to mental health services. The recent termination of government funding to the Spiritual Health Association (Holmes, personal communication, 18 March 2024) adds to this challenge, threatening the sustainability of spiritual care services in healthcare.

In a broader policy context, integrating spiritual care into healthcare systems has implications for both service delivery and resource allocation. Several leading psychiatric and psychological organisations have acknowledged the importance of spirituality and religion in mental health care. The World Psychiatric Association (WPA) issued a Position Statement on Spirituality and Religion in Psychiatry, which recommends that “a tactful consideration of patients’ religious beliefs and practices as well as their spirituality should routinely be considered and will sometimes be an essential component of psychiatric history taking” (Moreira-Almeida et al., 2016, p. 87). Similarly, Royal Australian and New Zealand College of Psychiatrists (RANZCP) has recognised the relevance of religion and spirituality to psychiatric practice. Their position statement notes that “psychiatrists should demonstrate respect for patients and their families by acknowledging their religious/spiritual beliefs and practices” and that “psychiatrists should be aware of the evidence base for psychiatric treatments which incorporate religious/spiritual principles” (Royal Australian and New Zealand College of Psychiatrists, 2018).

Although the economic case for spiritual care is underexplored, potential benefits include improved patient satisfaction, reduced mental health crises, and better patient outcomes (Best et al., 2020). In a recent Australian study, most participants expressed a need for including spiritual care in healthcare (Gordon et al., 2020). Other studies indicate that Australians wish to see their spiritual beliefs considered as part of healthcare (e.g. Best et al., 2014, 2015; Hilbers et al., 2010; Malviya, 2023). As mental health disorders continue to rise globally, with more than half of Australians expected to experience a mental health condition in their lifetime (ABS, 2022), addressing unmet spiritual needs may be one avenue to meet the growing demand for mental health services. Leveraging available resources, including spiritual care practitioners, aligns with the World Health Organization’s (WHO) vision of holistic mental healthcare (WHO, 2022) and could improve the cost-effectiveness of mental health interventions by integrating spirituality, a dimension intricately linked to mental well-being (Koenig et al., 2012).

By recognising spirituality as a part of mental healthcare, healthcare systems may better align with WHO’s vision for inclusive and holistic mental healthcare (WHO, 2022). Spiritual care practitioners can be a valuable resource in exploring spiritual dimensions in mental health assessments and providing spiritual care interventions. Their role may support inclusive, integrated, and person-centred mental healthcare (Best et al., 2020; WHO, 2022). However, their continued contribution to healthcare in Australia will likely depend on increased investment and support from both government and non-government organisations.

### Limitations

While this study explores the novel topic of the roles and contributions of spiritual care practitioners in mental healthcare, several limitations should be acknowledged. The findings are based on the perspectives of a small, convenient sample (n = 8), which limits the generalisability of the results. Although these insights are valuable, further research with larger and more diverse samples is needed to validate and expand upon the findings. Additionally, participants were recruited via the Spiritual Health Association (Victoria, Australia), which may have introduced selection bias. To gain a more comprehensive understanding of the contributions of spiritual care practitioners in mental health, future studies should incorporate perspectives from both mental health consumers and professionals.

## Conclusion

This study provides preliminary insights into the roles and contributions of spiritual care practitioners in mental health settings, offering a broad understanding of their work across diverse contexts. Findings reveal that spiritual care practitioners support holistic mental healthcare by providing person-centred, non-judgmental care, discerning between spiritual and psychotic experiences, and normalising clients’ spiritual narratives. Additionally, they educate mental health professionals about spiritual care, fostering a more inclusive approach within multidisciplinary teams. However, challenges such as limited professional recognition, funding, and respect from colleagues hinder their full integration. These findings add to the evidence base, emphasising the vital role of spiritual care practitioners and the need for future research on how best to support and integrate them into mental healthcare systems to improve care delivery and client outcomes.

### Key Findings

- Spiritual care practitioners play a crucial role in addressing clients’ spiritual needs by providing non-judgmental, person-centred care, tailored to clients’ unique spiritual beliefs and experiences.
- Spiritual care practitioners may assist in discerning between spiritual experiences and psychopathological symptoms, particularly in culturally diverse populations, helping prevent misdiagnosis.
- There is a need for increased professional recognition, funding, and acceptance of spiritual care practitioners within multidisciplinary mental health teams to ensure their full integration into mental health services.
